# Differential Flag Leaf and Ear Photosynthetic Performance Under Elevated (CO_2_) Conditions During Grain Filling Period in Durum Wheat

**DOI:** 10.3389/fpls.2020.587958

**Published:** 2020-12-18

**Authors:** Angie L. Gámez, Rubén Vicente, Rut Sanchez-Bragado, Iván Jauregui, Rosa Morcuende, Nieves Goicoechea, Iker Aranjuelo

**Affiliations:** ^1^Instituto de Agrobiotecnología, CSIC-Gobierno de Navarra, Mutilva, Spain; ^2^Instituto de Tecnología Química e Biológica António Xavier, Universidade NOVA de Lisboa, Oeiras, Portugal; ^3^Institute of Natural Resources and Agrobiology of Salamanca, IRNASA-CSIC, Salamanca, Spain; ^4^Department of Crop and Forest Sciences, University of Lleida – AGROTECNIO Center, Lleida, Spain; ^5^Plant Genetics, TERRA Teaching and Research Center, University of Liège, Gembloux, Belgium; ^6^Departamento Biología Ambiental, Grupo de Fisiología del Estrés en Plantas, Facultad de Ciencias (Unidad Asociada al CSIC, EEAD, Zaragoza, e ICVV, Logroño), Universidad de Navarra, Pamplona, Spain

**Keywords:** ear physiology, elevated CO_2_, durum wheat, photosynthesis acclimation, gene expression

## Abstract

Elevated concentrations of CO_2_ (CO_2_) in plants with C_3_ photosynthesis metabolism, such as wheat, stimulate photosynthetic rates. However, photosynthesis tends to decrease as a function of exposure to high (CO_2_) due to down-regulation of the photosynthetic machinery, and this phenomenon is defined as photosynthetic acclimation. Considerable efforts are currently done to determine the effect of photosynthetic tissues, such us spike, in grain filling. There is good evidence that the contribution of ears to grain filling may be important not only under good agronomic conditions but also under high (CO_2_). The main objective of this study was to compare photoassimilate production and energy metabolism between flag leaves and glumes as part of ears of wheat (*Triticum turgidum* L. subsp. *durum* cv. Amilcar) plants exposed to ambient [*a*(CO_2_)] and elevated [*e*(CO_2_)] (CO_2_) (400 and 700 μmol mol^–1^, respectively). Elevated CO_2_ had a differential effect on the responses of flag leaves and ears. The ears showed higher gross photosynthesis and respiration rates compared to the flag leaves. The higher ear carbohydrate content and respiration rates contribute to increase the grain dry mass. Our results support the concept that acclimation of photosynthesis to *e*(CO_2_) is driven by sugar accumulation, reduction in N concentrations and repression of genes related to photosynthesis, glycolysis and the tricarboxylic acid cycle, and that these were more marked in glumes than leaves. Further, important differences are described on responsiveness of flag leaves and ears to *e*(CO_2_) on genes linked with carbon and nitrogen metabolism. These findings provide information about the impact of *e*(CO_2_) on ear development during the grain filling stage and are significant for understanding the effects of increasing (CO_2_) on crop yield.

## Introduction

Atmospheric CO_2_ concentrations (CO_2_) have increased considerably since the pre-industrial era (≈280 ppm), reaching 414.5 ppm by 2020 (Noaa/Esrl, 2020). Estimated models for future greenhouse scenarios predict that CO_2_ concentration will not stabilize but will continue increasing over the coming decades (Ipcc, 2020). High (CO_2_) in plants with C_3_ photosynthetic metabolism, such as wheat, stimulate photosynthetic rates and consequently an increase in plant biomass is observed despite decreases in stomatal conductance (Aranjuelo et al., 2011). However, this response is not linear, and after the first stimulation, the photosynthetic rate tends to decrease as a function of the duration of high (CO_2_) exposure, which is due mainly to accumulation of sugars and down-regulation of photosynthetic machinery, including a decrease in Rubisco protein content and activity. This phenomenon is named photosynthetic acclimation (Aranjuelo et al., 2011; Dusenge et al., 2019). Leaf photosynthesis benefits from elevated CO_2_ by stimulating carboxylation of Rubisco, which is generally substrate limited (Farquhar et al., 1989), and by inhibiting the Rubisco oxygenation reaction, which leads to a loss of carbon (C) fixed during C_3_ photosynthesis (Long et al., 2004). The increases in leaf photosynthesis generate a remarkably enhanced production of different carbohydrates, which alters downstream processes (Ainsworth and Rogers, 2007). Moreover, low sink strength capacity appears to be related to photosynthetic down-regulation (Aranjuelo et al., 2011). Previous studies (Paul and Foyer, 2001; Borrill et al., 2015; Griffiths et al., 2016) have demonstrated that sink strength limits leaf photosynthesis. Thus, further work in understanding leaf and ear photosynthesis and C allocation is required to evaluate the ability of crops to exploit projected increases in atmospheric (CO_2_).

The ears are composed by awns, glumes, and ear bracts (lemma and paleae). During the last decade, significant effort has been put into understanding the photosynthetic contribution of entire ears and individual organ from ears, i.e., the glume, to grain filling in crops subjected to different growth conditions (Sanchez-Bragado et al., 2020 and references included). Recent evidence indicates that the contribution of ear photosynthesis to grain filling may be important not only under drought but also under good agronomic conditions (Maydup et al., 2010; Sanchez-Bragado et al., 2014a,b; Sanchez-Bragado et al., 2016). Although it has not been clearly demonstrated that ear photosynthetic rate under good agronomic conditions is higher than in the flag leaf lamina (Xu et al., 2009), a significant proportion of the C accumulated in wheat grains may originate in the ears (Aranjuelo et al., 2011; Sanchez-Bragado et al., 2014b; Zhou et al., 2014). In fact, ears exhibit better performance not only under water stress (Tambussi et al., 2007; Vicente et al., 2018) but also under high CO_2_ environments compared with the flag leaf, with the ear contributing more to grain yield (Zhu et al., 2008). Nevertheless, the net photosynthesis of the ear per unit one-sided organ area is lower than in the flag leaf lamina due to the presence of larger amounts of heterotrophic tissues in the ear. The capacity of ears to re-fix respired CO_2_ is twice the capacity of the flag leaf lamina (Araus et al., 1993; Bort et al., 1996; Gebbing and Schnyder, 2001; Tambussi et al., 2007, 2005; Vicente et al., 2018). Further, the xeromorphic anatomy of the ears (schlerophyllous traits of the bracts and particularly the awns) combined with a higher water use efficiency (Araus et al., 1993) and a greater osmotic adjustment (Tambussi et al., 2007, 2005) confer on the ears a higher relative water content compared to the rest of the plant (Morgan, 1980; Blum et al., 1988). Consequently, this mechanism to avoid dehydration in the ear results in delayed drought-induced or heat-induced senescence relative to the flag leaf lamina (Tambussi et al., 2007; Vicente et al., 2018). In addition, a longer developmental duration under high (CO_2_) environments has been observed in the ear compared to the flag leaf (Zhu et al., 2008).

A number of studies have revealed the important role of ears under different control and stressful growth conditions (Abebe et al., 2010; Jia et al., 2015; Sanchez-Bragado et al., 2014b, 2016, 2020). Although higher (CO_2_) are required to saturate photosynthesis in ears compared to the flag leaf in wheat (Knoppik et al., 1986), ear photosynthesis under elevated CO_2_ has been widely underestimated in the past (Teramura et al., 1990; Andre and DuCloux, 1993). Therefore, recent studies have sought to estimate the role of ears under elevated CO_2_ by deploying Free-Air CO_2_ Enrichment (FACE) (Wechsung et al., 2000; Zhu et al., 2008, 2009) and semi-controlled conditions (Li et al., 2019). In addition, molecular markers associated with morphological traits of the ear have been identified in wheat (Sourdille et al., 2002; Molero et al., 2019) and assessment of the key metabolic profiles of ear bracts has also been undertaken in a non-destructive manner using spectroradiometers (Vergara-Diaz et al., 2020). However, until recently, studies to estimate the photosynthetic performance of the ear that have combined analyses at the physiological, metabolomic, and transcriptomic level have been scarce, especially in high CO_2_ environments. Nonetheless, evidence from integrative analysis of the transcriptomic and phenotypic responses of the ear and the flag leaf in wheat has been reported by few authors under ambient CO_2_ (Vicente et al., 2018) and high CO_2_ conditions (Aranjuelo et al., 2008, 2011). Despite the response of leaf photosynthesis to elevated CO_2_ having been detailed broadly, there has been little attention given to the photosynthetic performance of ears and other organs, such as glumes, under high (CO_2_) in wheat. Therefore, the objective of this study was to analyze the impact of elevated atmospheric CO_2_ on the photosynthetic machinery of wheat flag leaves and ears. For this purpose, we have characterized the concentration of target metabolites transcript levels of C and N metabolism genes, as well as other physiological parameters in both organs at grain filling stage.

## Materials and Methods

### Plant Material and Experimental Design

The experiment was conducted with durum wheat plants (*Triticum turgidum* subsp. *durum*), using the genotype Amilcar, which is a commercial wheat cultivar widely used in the Navarra region. Wheat seedlings were vernalized for 1 month (during January 2017) at 4°C and then transplanted into 13 L pots with six plants per pot, containing a mixture of 2:2:1 of vermiculite:perlite:peat. The plants were grown at the University of Navarra campus (42°80′N, 1°66′W; Pamplona, Spain), at average ambient temperature of 20°C and 50% relative humidity. The study was conducted in four separated greenhouses with doors along the longitudinal walls to allow the access to plants inside, two greenhouses kept at ambient air CO_2_ concentration [*a*(CO_2_)] and the other two at elevated CO_2_ [*e*(CO_2_)]. In *a*(CO_2_) conditions no CO_2_ was added and CO_2_ was close to 400 ± 20 μmol mol^–1^. In the *e*(CO_2_) treatment, the (CO_2_) was close to 700 ± 20 μmol mol^–1^ (24 h per day) by injecting pure CO_2_ from the start of the experiment. The (CO_2_) was continuously monitored using a Guardian Plus gas monitor (Edinburgh Instruments Ltd., Livingston, United Kingdom) whose signal controls the valve that injects the CO_2_. The CO_2_ used to increase CO_2_ level was provided by Air Liquide (Valladolid, Spain). The isotopic composition (δ^13^C) within the elevated CO_2_ level modules was −20.6‰ whereas in case of ambient CO_2_ greenhouses δ^13^C values were −10.3‰. The pots were located separately in the middle of each greenhouse and they were rotated weekly to avoid edge effects. There were eight replicates (pots) in each treatment distributed in two greenhouses with the same (CO_2_) concentration. The plants were surrounded by additional pots with in order to avoid edge effect, with an average distance of 5 cm between pots. Plants were watered with a complete Hoagland nutrient solution twice a week and with water once a week until harvesting.

Measurements of gas exchange and sampling to determination of carbon – C- isotope composition, metabolic analysis and gene expression were conducted when plants reached 2 weeks after anthesis (Zadoc stage 65). This period frequently is more sensitive to environmental changes because coincides with the largest photoassimilate contribution to grain filling (Aranjuelo et al., 2011). Determinations and sampling were carried out 2–5 h after sunrise. The flag leaves and ears collected were plunged immediately into liquid nitrogen and stored at −80°C for further analyses. The glumes were separated from the ears under liquid nitrogen.

### Agronomic Determinations

When the plants reached the maturity stage, plant shoot, ears, and grains were collected. For this purpose, eight plants were harvested for each treatment combination. Collected samples were weighed after drying in an oven at 60°C during 48 h. Total dry matter (DM) was determined and included ear DM and shoot DM. Further, the agronomical parameters, thousand kernel weight (TKW), harvest index (HI = ratio between seed weight and total DM) and grain number were determined.

### Gas Exchange Determinations

Gas exchange measurements were conducted 2 weeks after anthesis in the central segment of the flag leaves and complete ears of plants grown under both (CO_2_) treatments. The net (*An*) and apparent gross photosynthesis – *A*(gross), stomatal conductance (*gs*), sub-stomatal CO_2_ mole fraction (*Ci*) and electron transport rate (ETR) were determined at 25°C for each growth condition using a Li-Cor 6400 XP portable gas exchange photosynthesis system (LI-COR, 6400 XT, United States), with a photosynthetic photon flux density (PPFD) of 1200 μmol m^–1^ s^–1^. Photosynthesis was recorded at 400 and 700 μmol mol^–1^ in each treatment. To taking into account the respiration rates, the sum between *An* plus dark respiration (*Rd*) (*An* + *Rd*) was defined as *A*(gross). To conduct dark respiration measurements the plants were dark-adapted for 4 h. Maximal carboxylation velocity by Rubisco (*Vc*_*max*_) was determined using the method of Harley et al. (1992). To measure ear gas exchange, entire ears were placed in a homemade aluminum chamber (20 × 12 × 6 × 10^–6^ m^3^) connected to the LiCOR 6400 XT gas exchange analyzer (Aranjuelo et al., 2011). Ear surface was determined in 2D area basis through a scanner. Within the chamber, ingoing air was passed through the chamber at a rate of 1 L min^–1^. The molar fractions of CO_2_ and humidity were measured with the infrared gas analyzer of the LiCOR-6400XT. The CO_2_ partial pressure was maintained constant with the infrared gas analyzer-controlled CO_2_ injection system. To ensure steady-state conditions inside the chamber, the system was left to stabilize for a few minutes. An external light source composed of LED lights was placed around the chamber during the measurements, achieving a saturating PPFD of approximately 1200 μmol m^–2^ s^–1^ inside the chamber.

### Carbon Isotope Composition Analysis

The stable C isotope composition (δ^13^C) was conducted 2 weeks after anthesis in leaves and glumes separated manually from the ear. For δ^13^C analysis of DM, approximately 1 mg of each sample was weighed into tin capsules and measured with an elemental analyzer coupled to an Isotope Ratio Mass Spectrometer (Delta C IRMS, ThermoFinnigan, Bremen, Germany) operating in continuous flow mode in order to determine the stable C (^13^C/^12^C) isotope ratios of the same samples. The ^13^C/^12^C ratios of plant material were expressed in δ notation (Coplen, 2008):

δ13⁢C=(C13/C12)sample(C13/C12)standard-1,

where “sample” refers to plant material and “standard” to international secondary standards of known ^13^C/^12^C ratios (IAEA CH7 polyethylene foil, IAEA CH6 sucrose and USGS 40 L-glutamic acid) calibrated against Vienna Pee Dee Belemnite calcium carbonate (VPDB) with an analytical precision (SD) of 0.10‰.

### Sugar and Organic Acid Contentsc

For sugar extraction, dried flag leaves and glumes were ground to a fine powder. About 25 mg of the powder were suspended and mixed in 1 ml of ethanol (80%) in an Eppendorf tube, then the samples were shaken in a thermomixer (90 min, 70°C, 1100 rpm) and centrifuged at 20,800 × *g* for 10 min at 22°C. The pellet was used to starch quantification and the supernatant was used for soluble sugar quantification. To solubilization of starch, the pellet was re-suspended in KOH 0.2N and starch extraction was done using an amyloglucosidase test kit (R-Biopharm AG, Darmstadt, Germany). Finally, the quantification was performed through absorbance measurements at 340 nm. Glucose, fructose and glucose were determined in the supernatant obtained from flag leaves, glumes and grain organs using an ionic chromatographer (ICS-3000, Thermo Scientific^TM^, United States).

The organic acids (OAs) malate, citrate, oxalate, and succinate were determined using isocratic ion chromatography in the supernatant fraction after ethanol (80% v/v) extraction for 25 min at 30°C in an ultrasonic bath. Subsequently, the extracts were filtered with Milex filters (Millipore, Billerica, MA, United States) and injected into a DIONEX-DX500 (Dionex Corporation, CA, United States) system equipped with an ED40 electrochemical detector. An IonPac AS11 column connected to an ATC-1 protecting column and an AG11 pre-column (Dionex, Salt Lake City, UT, United States) was used.

### Nitrogen Concentration, Total Amino Acid and Soluble Protein Contents

N concentration and total amino acid content (TAA) were determined in flag leaves, glumes, and grains from plants grown under *a*(CO_2_) and *e*(CO_2_). N elemental content was determined in dry and ground material at 60°C for 48 h based on sample dynamic combustion, using an elemental analyzer (EA1108, Carbo Erba Instrumentazione, Veneto, Italy). Total amino acids were determined in lyophilized, ground and weighed (≈20 mg) samples. The ground material was suspended in 500 μl of ethanol (80% v/v), shaken in thermomixer (60 min, 80°C, 600 rpm) and centrifuged (10 min, 4°C, 14,000 × *g*). After centrifugation, the supernatant was collected and the ethanol was evaporated at *Speed vacuum* for 3 h. The residue obtained is re-suspended in 100 μl of deionized water and centrifuged. The supernatant was used to derivatization with an ACCQ-Fluor^TM^ Reagent kit (WATER, United States) based on borate buffer, acetonitrile and AQC derivatizing reagent (6-aminoquinolyl-N-hydroxysuccinimidyl carbamate) using High Performance Liquid Chromatography (HPLC). The total soluble protein (TSP) concentration was also measured in these samples by the Bradford method (Bradford, 1976).

### Quantitative Real-Time PCR Analysis

RNA from 100 mg of frozen plant material (flag leaves and glumes) was isolated as described by Morcuende et al. (1998). A 10 μg quantity of RNA per sample was treated with Turbo DNase (Ambion) according to the manufacturer’s instructions, and subsequently the absence of genomic DNA was tested by PCR using a primer pair for the *PolA1* gene (AB647308) as described in Vicente et al. (2015). Afterward, RNA integrity was checked on a 1.5% (v/v) agarose gel prior to and after DNase digestion. cDNA was synthesized using SuperScript III reverse transcriptase (Invitrogen GmbH) according to the manufacturer’s instructions. Only cDNA preparations that yielded similar C_*t*_ values for the reference gene were used for comparing transcript levels of target genes. Polymerase chain reactions were performed in a 384-well optical plate using a sequence detection system (ABI PRISM 7900 HT, Applied Biosystems), with SYBR Green PCR Master Mix (Applied Biosystems), diluted cDNA (1:40) and the specific primers of each gene (Vicente et al., 2015). The thermal profile was as follows: 50°C for 2 min, 95°C for 10 min, 40 cycles of 95°C for 15 s and 60°C for 1 min, and a final step of 95°C for 15 s and 60°C for 15 s to obtain the dissociation curve. Three biological replicates were used for quantification analysis and two technical replicates were analyzed for each biological replicate.

The gene encoding ADP-ribosylation factor (Unigene Ta2291) was used as a reference gene for normalizing gene expression results after validating its expression stability in our growth conditions, in agreement with Vicente et al. (2015). We evaluated the transcript levels for 30 genes associated with photosynthesis, carbohydrate metabolism, glycolysis, the tricarboxylic acid (TCA) cycle and N assimilation in flag leaves, and glumes using the primers described in Vicente et al. (2015). The specificity of PCR amplification was confirmed by the presence of unique amplicons of the expected length on 3.5% (w/v) agarose gels. All genes analyzed and their abbreviations are shown in Supplementary Table 1. The values of the cycle threshold (C_*t*_) were calculated using SDS 2.4 software (Applied Biosystems). Relative gene expression was quantified using the comparative Ct method 2^–ΔΔCt^ (Schmittgen and Livak, 2008), and the data were presented as the log_2_ ratio of *e*(CO_2_) over *a*(CO_2_) for each organ.

### Statistical Analysis

Statistical analyses for the effects of *e*(CO_2_) relative to *a*(CO_2_) and/or the differences between organs (flag leaves and ears or glumes) were performed through one- and two-factor analyses of variance (ANOVA) in R environment (R^®^ v.3.4.2, 2017; Boston, MA–Seattle, WA, United States). Differences between *a*(CO_2_) and *e*(CO_2_) treatments means and/or organs were assessed using Tukey’s HSD test. Significance were accepted at *p* < 0.05. All figures were generated in Sigma-Plot 11.0 program (Systat Software Inc.).

## Results

### Growth and Physiological Parameters

Plant growth data (Table 1) showed that exposure to *e*(CO_2_) increased plant growth. Such increase was linked to an stimulation in grain yield (explained by an enhancement in grain number) of *e*(CO_2_).

**TABLE 1:** Effect of *a*(CO_2_) and *e*(CO_2_) (400 vs. 700 μmol mol^–1^) on shoot, ear, total and grain biomass, thousand kernel weight (TKW), harvest index (HI), and grain number of durum wheat plants.

CO_2_	Shoot DM	Ear DM	Total DM	Grain DM	TKW	HI	Grain number
	
	G						
*a*(CO_2_)	12.70 ± 0.67	10.50 ± 0.82	24.72 ± 1.72	6.38 ± 0.38	47.69 ± 3.44	0.26 ± 0.00	141.92 ± 23.40
*e*(CO_2_)	16.63 ± 0.90	17.97 ± 1.23	34.60 ± 1.66	11.81 ± 1.09	40.09 ± 2.43	0.34 ± 0.02	298.60 ± 30.15
*p*-Value	0.006	**0.001**	**0.002**	**0.001**	0.101	0.006	**0.002**

*Each value represents the mean ± SE (*n* = 3–4). The *p*-value in bold means significant differences between (CO) in each organ.*

**FIGURE 1:**
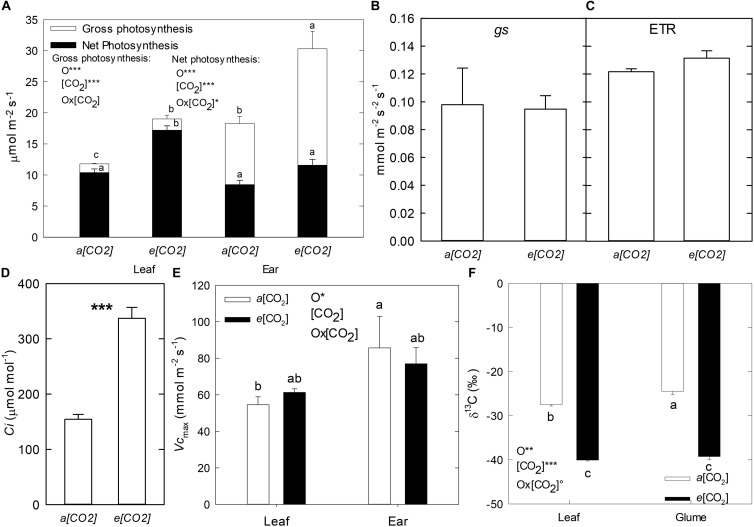
Effect of *a*(CO_2_) and *e*(CO_2_) (400 vs. 700 μmol mol^– 1^, respectively) on **(A)** Gross and net photosynthesis, **(B)** stomatal conductance (*gs*), **(C)** electron transport rate (ETR), **(D)** Intercellular (CO_2_) (*Ci*), **(E)** Rubisco maximum carboxylation (*Vc*_*max*_) in flag leaf and ear and **(F)** Isotopic composition ^13^C (δ^13^C) in flag leaf and glume of durum wheat plants. Each value represents the mean ± SE (*n* = 3–4). Different letters indicate significant differences (*p* < 0.05). Asterisks indicate significant differences: °*p* < 0.1; *p < 0.05; **p < 0.01; ***p < 0.001. O, organ; (CO_2_), CO_2_ concentration (elevated/ambient); O*(CO_2_), interaction between organ and CO_2_ concentration.

In order to compare the physiological response to *e*(CO_2_) between the flag leaf and ear, the gas exchange parameters were measured (Figure 1). Mean values of apparent gross photosynthesis – *A*(gross) were higher under *e*(CO_2_) compared to *a*(CO_2_) in both flag leaves and ears, being 61 and 66% greater, respectively. However, *A*(gross) was higher in the ear compared to the flag leaf, due mainly to a higher proportion of dark respiration (*Rd*) (Figure 1A). In addition, in the ear the *Rd* was greater than net photosynthesis (*An*) by a 118% under *a*(CO_2_) and 164% under *e*(CO_2_) conditions, while in the flag leaf the *Rd* was lower than *An* with only 20 and 8% under *a*(CO_2_) and *e*(CO_2_), respectively. No significant differences were found between (CO_2_) treatments for *gs* and ETR in flag leaves (Figures 1B,C). In contrast, the intercellular CO_2_ mole fraction (*Ci*) in flag leaves was considerably higher under *e*(CO_2_) than *a*(CO_2_) (Figure 1D). Moreover, the magnitude of the CO_2_ gradient (*Ca*-*Ci*, *Ca:* Concentration of CO_2_ at ambient) was stronger under *e*(CO_2_) (362.8 μmol mol^–1^) than *a*(CO_2_) (245.7 μmol mol^–1^) which would affect the isotope discrimination. Although no differences between CO_2_ conditions were observed in *Vc*_*max*_, at the organ level it was higher in ears than flag leaves (Figure 1D). The C isotopic composition (δ^13^C) decreased under *e*(CO_2_) compared to *a*(CO_2_) for both flag leaves and glumes (46 and 60%, respectively). However, the leaves showed lower δ^13^C values (more negative) than glumes under *a*(CO_2_) (Figure 1F).

### Total Soluble Carbohydrates and Starch Content

The total soluble carbohydrates (TSCs), which comprise the sum of glucose, fructose, and sucrose concentrations, tended to be higher in plants grown under *e*(CO_2_) (Table 2) irrespective of the organ evaluated. In comparison to the glumes and grains, the flag leaves showed a greater increase in TSCs [c.a. 136% at *e*(CO_2_) rather *a*(CO_2_)]. Among the individual sugars, glucose was not significantly different between the CO_2_ conditions, but fructose increased under *e*(CO_2_) in leaves (225%) and grains (176%) and decreased in glumes (49%) relative to *a*(CO_2_). The sucrose content at *e*(CO_2_) increased significantly in leaves (146%), decreased in grains (63%) but was not significantly different in glumes. The starch content increased in glumes and grains under elevated CO_2_ (88 and 5%, respectively).

**TABLE 2:** Effect of *a*(CO_2_) and *e*(CO_2_) (400 vs. 700 μmol mol^–1^) on glucose, fructose, sucrose, total soluble carbohydrates (TSCs) and starch contents in flag leaf, glume, and grain of durum wheat plants.

Organ	CO_2_	Glucose	Fructose	Sucrose	TSC	Starch
		
		mg g^–^^1^ DM				
Flag leaf	Ambient CO_2_	11.82 ± 0.51	8.37 ± 0.66	71.39 ± 4.97	91.58 ± 4.52	4.96 ± 0.09
	Elevated CO_2_	13.20 ± 5.93	27.21 ± 6.54	175.70 ± 10.58	216.11 ± 14.58	4.37 ± 0.22
	*p*	0.828	**0.046**	<**0.001**	**0.001**	0.066
Glume	Ambient CO_2_	1.54 ± 0.32	3.13 ± 0.18	4.21 ± 0.66	8.88 ± 0.76	1.47 ± 0.08
	Elevated CO_2_	3.47 ± 0.84	1.60 ± 0.43	7.47 ± 2.03	12.54 ± 3.16	2.77 ± 0.11
	*p*	0.098	**0.031**	0.202	0.323	<**0.001**
Grain	Ambient CO_2_	100.67 ± 4.18	46.17 ± 2.84	110.51 ± 8.30	257.34 ± 13.91	2.67 ± 0.04
	Elevated CO_2_	117.61 ± 9.33	127.39 ± 16.87	40.53 ± 0.95	285.54 ± 20.64	2.81 ± 0.02
	*p*	0.173	**0.009**	**0.001**	0.321	**0.041**
	Organ (O)	***	***	***	***	***
*Significance*	(CO_2_)	*	***	*	***	**
	Organ*(CO_2_)	ns	***	***	***	***

Each value represents the mean ± SE (*n* = 3–4). The *p*-value in bold means significant differences between (CO_2_) in each organ. Levels of significance: ns, not significant; *p < 0.05; **p < 0.01; ***p < 0.001.

### Organic Acid Contents

**FIGURE 2:**
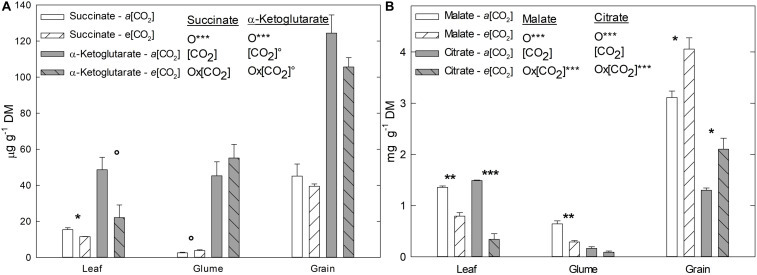
Effect of *a*(CO_2_) and *e*(CO_2_) (400 vs. 700 μmol mol^– 1^, respectively) on **(A)** succinate, α-ketoglutarate (μg g^– 1^ DM), **(B)** malate and citrate (mg g^– 1^ DM). Each value represents the mean ± SE (*n* = 3–4). Asterisks indicate significant differences: °*p* < 0.1; *p < 0.05; **p < 0.01; ***p < 0.001. O, organ; (CO_2_), CO_2_ concentration (elevated/ambient); O*(CO_2_), interaction between organ and CO_2_ concentration.

In order to further compare the response of C and/or energy metabolism under *e*(CO_2_), four C-rich OAs related to the TCA cycle and other metabolic pathways were analyzed in flag leaves, glumes, and grains, namely succinate, malate, α-ketoglutarate, and citrate (Figure 2). Their contents, designated here as total OA content, tended to decrease under *e*(CO_2_) in flag leaves and glumes. Significantly, succinate, malate, and citrate in leaves and malate in glumes were lower under *e*(CO_2_), although the malate and citrate contents were higher in grains (Figure 2B). At the organ level, for all OAs measured, grains had the highest values relative to leaves and glumes, while, glumes had the lowest content compared to the other organs, except for α-ketoglutarate (Figure 2A).

### Nitrogen Concentration, Total Amino Acid and Total Soluble Protein Contents

**FIGURE 3:**
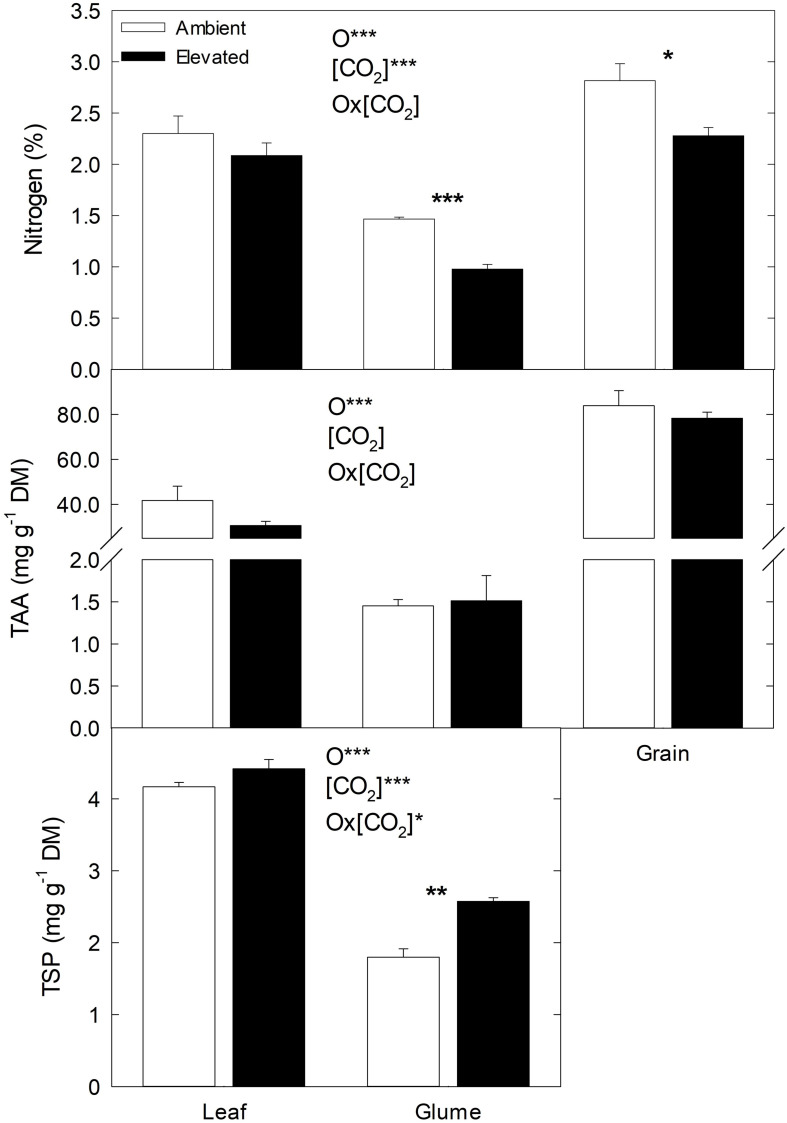
Effect of *a*(CO_2_) and *e*(CO_2_) (400 vs. 700 μmol mol^– 1^, respectively) on N content (%), total amino acids (TAA, mg g^– 1^ DM) and total soluble protein (TSP, mg g^– 1^ DM). Each value represents the mean ± SE (*n* = 3–4). Asterisks indicate significant differences: *p < 0.05; **p < 0.01; ***p < 0.001. O, organ; (CO_2_), CO_2_ concentration (elevated/ambient); O*(CO_2_), interaction between organ and CO_2_ concentration.

In general, N concentration and TAA content were lower under *e*(CO_2_) than *a*(CO_2_), while TSP content was higher (Figure 3). In comparison to leaves and glumes, N and TAA contents were higher in grains than leaves and glumes, while TSP content was higher in leaves than glumes. Although N and TAA contents tended to decrease and TSP to increase under *e*(CO_2_) in leaves, the differences were not statistically significant. Nevertheless, the effect of *e*(CO_2_) was remarkable in glumes; in this organ, the N decreased 33% and TSP increased 43% under *e*(CO_2_) compared to *a*(CO_2_). In grains, N decreased 19% under *e*(CO_2_) and no effects were found in the TAA content at different CO_2_ concentrations.

### Relative Gene Expression in Flag Leaves and Glumes

**FIGURE 4:**
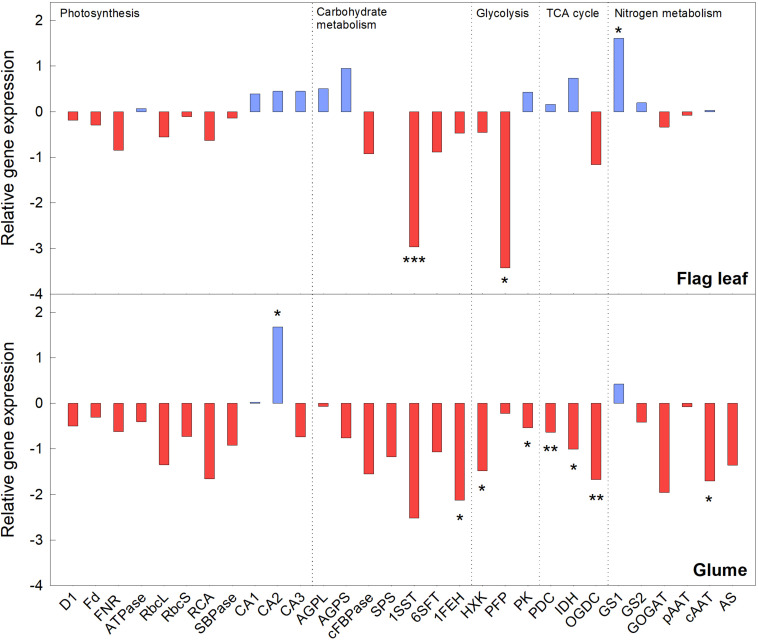
Relative transcript levels of durum wheat plants exposed to *e*(CO_2_) in flag leaf and glume organs. Asterisks indicate a significant difference between (CO_2_) treatments: *p < 0.05; **p < 0.01; ***p < 0.001.

In this study, the relative expression of genes linked to photosynthesis, carbohydrate metabolism, glycolysis, the TCA cycle, and N metabolism were analyzed to study the effect of *e*(CO_2_) over *a*(CO_2_) for each organ. In general, most of the genes analyzed were substantially down-regulated under *e*(CO_2_) in flag leaves and glumes. Two genes linked to carbohydrate metabolism (sucrose:sucrose 1-frutosyltransferase, *1SST*) and glycolysis (pyrophosphate-fructose-6-phosphate 1-phosphotransferase, *PFP*) were significantly down-regulated under *e*(CO_2_) in flag leaves, while other genes related to light harvesting and the Calvin–Benson cycle tended to decrease. Cytosolic glutamine synthetase (GS1) gene expression was significantly up-regulated in flag leaves under *e*(CO_2_), and the expression of other genes related to respiration, starch synthesis and carbonic anhydrases tended to increase (Figure 4). On the other hand, there was greater repression of transcript levels in the glumes than the flag leaves under *e*(CO_2_), indicating a general repression of glume metabolism. While the plastidial carbonic anhydrase (*CA2*) was significantly up-regulated in *e*(CO_2_), the genes related to sugar degradation and respiration (fructan 1-exohydrolase, *1FEH*; hexokinase, *HXK*; pyruvate kinase, *PK*; E1 component α-subunit of mitochondrial pyruvate dehydrogenase complex, *PDC*; mitochondrial NAD-dependent isocitrate dehydrogenase, *IDH*; and E1 subunit of 2-oxoglutarate dehydrogenase complex, *OGDC*), and N metabolism (cytosolic aspartate aminotransferase, *cAAT*) were significantly down-regulated.

## Discussion

### Elevated CO_2_ Alters Gas Exchange, Carbon and Nitrogen Metabolism

It is well documented that in C_3_ plants *e*(CO_2_) increases leaf photosynthesis and reduce stomatal conductance (Ainsworth and Rogers, 2007), which in absence of soil water or nitrogen limitations, increases plant biomass, and yield (Reich et al., 2014; Gray et al., 2016). However, little is known about other photosynthetic organs, as earns. The current study showed that, in agreement with above mentioned studies, the net (*An*) and apparent gross photosynthesis – *A*(gross) exhibited higher values at *e*(CO_2_) in both flag leaves and ear organs compared to *a*(CO_2_), which contributed to increase grain yield and plant biomass of plants under *e*(CO_2_). The stimulation of *An* and plant growth under *e*(CO_2_) conditions has been documented by several authors (Zhu et al., 2008; Aranjuelo et al., 2015; Dusenge et al., 2019). In additional physiologic analyses, did not detect differences in *gs* and ETR at *e*(CO_2_) in flag leaves, but *Ci* increased under *e*(CO_2_) with a strong gradient of CO_2_ (Figure 1). These results indicate that the enhancement of *An* by *e*(CO_2_) may be caused predominantly by the increase in (CO_2_) at the site of CO_2_ fixation, which increases the CO_2_/O_2_ ratio, thus promoting the efficiency of Rubisco carboxylation in leaf tissues (Gamage et al., 2018). Further, it should be also considered that due to the fact that under *e*(CO_2_) air is enriched in ^12^CO_2_, this would contribute to increase the absolute concentration of ^12^CO_2_ at the active site. In spite of changes in δ^13^C due to the CO_2_ treatment, there were differences associated with each organ. The discrimination of ^13^C depends on the ratio of the assimilation rate to CO_2_-diffusive conductance (Sanchez-Bragado et al., 2014a). In our study, the glumes showed higher values of δ^13^C than the flag leaves, at least under *a*(CO_2_). This could be due to lower permeability to gas diffusion in the ears compared to leaves, which caused the increase in δ^13^C in this organ (Sanchez-Bragado et al., 2014a). However, no differences in δ^13^C were observed in the flag leaves and glumes at *e*(CO_2_), which there is enough ^12^C supply.

Additionally, plant respiration rates (*Rd*) may be altered under *e*(CO_2_) conditions (Gamage et al., 2018). To distinguish the differential effect of *e*(CO_2_) in respiration of flag leaf and ear, the *A*(gross) was measured. In this study, the gross photosynthesis increased in the ears (Figure 1A) due to higher respiration rates compared to the flag leaves at *e*(CO_2_). Previous studies have reported greater *Rd* values than *An* in the ear, as a consequence of the respiration of developing grains and non-photosynthetic tissues (Araus et al., 1993). The fact that under elevated (CO_2_) grain DM and carbohydrate content increased, would help to explain the larger respiration rates of those plants. Studies in ears of cereals with C_3_ photosynthesis has suggested that the high levels of CO_2_ released by the respiration of the developing grains can be re-fixed in the ear itself (Tambussi et al., 2007; Sanchez-Bragado et al., 2020). This re-fixation capacity might confer an important role on the ears of providing assimilates for grain filling (Araus et al., 1993; Bort et al., 1996; Tambussi et al., 2007; Sanchez-Bragado et al., 2014a; Zhou et al., 2014; Vicente et al., 2016). Zhu et al. (2009) suggested that the role of the ears to supply assimilates during grain-filling could be larger under *e*(CO_2_) conditions due to the acceleration of senescence in flag leaves compared to the ears.

As consequence of the increase in *An* at *e*(CO_2_) an accumulation of sugars takes place (Zhu et al., 2009; Watanabe et al., 2014; Aranjuelo et al., 2015; Vicente et al., 2015; Dusenge et al., 2019), as observed in the current study for fructose, sucrose and TSCs (Table 2). However, sugar accumulation was more substantial in flag leaves than glumes, which may be due to reduced or insufficient sink capacity in the ears and grains under these conditions (Leakey et al., 2009). The fact that glumes are “closer” to grains (target sink) could also affect the carbohydrate balance of the ear. Within this context, it should be also considered that, the changes in metabolic compounds, as sugars, are dynamics respect the time of the day and can be modified constantly according the environmental conditions. In this sense, *e*(CO_2_) can alter the C source-sink balance in the plants (Fabre et al., 2019). For this reason, in the present study we consider a transitory change and/or accumulation of metabolites, such as the sugars, and the results represent only a unique time of the day. Having this assumption in mind, while the starch content tended to decrease in leaves, it increased in glumes and grains, possibly as a strategy to release soluble sugars and translocate them away from leaves, with a consequent reduction in starch accumulation (Gamage et al., 2018). Furthermore, as mentioned above, high concentrations of sugars can be related to the stimulation of respiration, as observed in Figure 1A, with the sugars acting as substrates for respiration (Dusenge et al., 2019), especially in the ears. Nevertheless, carbohydrate accumulation it is not the only factor that alters respiration rate, various intermediates of the respiration system, such as OA, also have a similar influence. Thus, changes in OA availability might affect respiration rates under *e*(CO_2_). In our study, the OA content tended to decrease at *e*(CO_2_) (Figure 2), which contrasts with increases that have been reported for the day period (Watanabe et al., 2014). Interestingly, Sweetlove et al. (2010) indicated that OA may be consumed as a respiratory substrate even in the light, highlighting that the TCA cycle is not only important at night. Moreover, increases in citrate levels, especially in grains, can serve as C skeletons for the synthesis of N-rich compounds, such as amino acids and proteins (Watanabe et al., 2014).

In this study, the N and TAA content decreased in flag leaves, glumes and grains of plants grown under *e*(CO_2_), in agreement with earlier reports in durum (Aranjuelo et al., 2015; Vicente et al., 2015) and spring wheat (Lopes et al., 2006; Del Pozo et al., 2007; Andrews et al., 2019). Such a decline in N concentration can be explained by a dilution of N in tissues due to increase in C assimilation (Vicente et al., 2015), reduction in plant N demand (Stitt and Krapp, 1999), a decrease in N assimilation (Bloom, 2015) related to the inhibition of leaf primary assimilation (Vicente et al., 2016) and/or photorespiration (Bloom et al., 2012), and acceleration of leaf N remobilization (Aranjuelo et al., 2015). Our results showed an increase in TPS content under *e*(CO_2_), which can be associated with a reduction in amino acid content due to its utilization in protein synthesis (Lopes et al., 2006). Moreover, the differences between CO_2_ treatments for N and TSP content were significant in glumes, but not in flag leaves (Figure 3). This could have been due to the flag leaves undergoing an accelerated senescence under *e*(CO_2_), which causes a greater N translocation from leaf to grain to support the N demand during grain filling (Zhu et al., 2009), with considerable depletion in N content in glumes. Our results support earlier observations that the glumes have a prominent role in N feeding of the grain (Lopes et al., 2006).

### Photosynthesis Acclimation Confirmed by Gene Expression

The initial stimulation of photosynthetic rate in C_3_ plants under *e*(CO_2_) is well characterized and determined in the current study. However, it has been previously observed that photosynthesis decreases in response to prolonged exposure to *e*(CO_2_) (Martínez-Carrasco et al., 2005; Del Pozo et al., 2007; Aranjuelo et al., 2011, 2015; Vicente et al., 2015). This phenomenon is known as “photosynthetic acclimation” and it is characterized by the suppression of several genes expression (Gamage et al., 2018). The present study analyzed the relative gene expression of photosynthesis, carbohydrate metabolism, glycolysis, TCA cycle and N metabolism in both flag leaf and glume organs. In agreement with previous manuscript, our study showed that the expression level of most of genes related to photosynthesis [such as Rubisco, Rubisco activase, and Ferredoxin-NADP(H) oxidoreductase] tended to be down-regulated in leaf and glume tissues under *e*(CO_2_) (Figure 4). Previous studies have reported the down-regulation of genes encoding for both small and large sub-unit (*rbc*S and *rbc*L) at *e*(CO_2_) (Bokhari et al., 2007; Aranjuelo et al., 2011; Takatani et al., 2014; Vicente et al., 2015) and this declination is often associated to the increase in carbohydrate content and lower plant N concentrations (Vicente et al., 2016, 2015). Furthermore, in the current study the relative gene expression of carbonic anhydrases (*CA1* and *CA2*) was up-regulated under *e*(CO_2_) relative to *a*(CO_2_), and mainly in the glumes (Figure 4). These enzymes catalyze the reversible conversion of CO_2_ to HCO_3_, and have been related to the regulation of mesophyll conductance (Evans et al., 2009). Our results resembled previous findings (Aranjuelo et al., 2011), suggesting that the up-regulation in expression of those genes under *e*(CO_2_) might ensure the supply of CO_2_ to the chloroplasts, due to the increase in *Ci* and gradient *Ca*-*Ci* under such conditions.

The *e*(CO_2_) induced the carbohydrate accumulation in leaves and glume. We observed a down-regulation of the expression of genes related to carbohydrate metabolism in plants grown in *e*(CO_2_), while the sugars increased. The accumulation under such conditions can be associated to the repression of genes related to carbohydrate degradation, such as Fructan 1-exohydrolase (*FEH)*, as reported in durum wheat (Vicente et al., 2015). A similar pattern of changes was also found in the expression of the *FEH* gene in glumes. The repression of gene expression of glycolysis and TCA enzymes, mainly in glumes, was observed in this study despite the increase in *Rd*, it can be due to the carbohydrate accumulation at *e*(CO_2_) (Markelz et al., 2014). These results are consistent with Vicente et al. (2015) and Markelz et al. (2014). Another relevant issue within this context is the close interaction among TCA, C, and N metabolism. Several TCA intermediaries (such as malate, α-oxoglutarate) are required to regulate the N assimilation and amino acid biosynthesis, mainly asparagine, aspartic acid, glutamine, and glutamic acid, in responses of changes of C metabolism (Morcuende et al., 1998). In general, these intermediaries provide C skeletons for N assimilation (Watanabe et al., 2014). Taking this into account, the repression of N metabolism genes observed in glumes at *e*(CO_2_) could be associated to repression of TCA genes as precursors of amino acids synthesis. Finally, the fact that, in *e*(CO_2_) glumes, total protein was increased could be also reflecting fast assimilation of amino acids in proteins in those plants. The slight increase in GS1 would contribute to explain such response. In case of flag leaves, the strong up-regulation on GS1 would remark that was up-regulated (Lopes et al., 2006) under *e*(CO_2_) in flag leaves, which could indicate an assimilation of N in proteins. The decrease in the expression of most of the genes related to primary assimilation in glumes, together with the low N levels and amino acid content at *e*(CO_2_) suggested a fast remobilization during grain filling, as reported in durum wheat by Aranjuelo et al. (2015).

## Conclusion

Our results indicate a differential response of ears relative to flag leaves in durum wheat plants grown under *e*(CO_2_). The higher ear gross photosynthesis, together with leaf photosynthesis enhancement, explained the increase in plant biomass and yield under *e*(CO_2_). The current study also showed that, in general, ears showed higher apparent gross photosynthesis and respiration rates that contributed to increase plant biomass. However, *e*(CO_2_) plants also showed lower accumulation of soluble sugars, apparently faster N remobilization and assimilation, and stronger repression of genes compared to the flag leaf. Our findings also support the concept that photosynthetic acclimation to *e*(CO_2_) was driven by accumulation of carbohydrates, a reduction in the N concentration and repression of genes related to photosynthesis, glycolysis, the TCA cycle and the N assimilation pathway, with the changes in gene expression being more pronounced in glumes than leaves. This highlights the importance of investigating the close interaction between C and N metabolic pathways at the biochemical and transcriptional level in the different organs that contribute to grain development, and under the conditions anticipated for future changes in climate. Further, the relevance of N availability on adjustment of C metabolism (and crop yield) is a major issue that should be considered. This study provides much needed information on how *e*(CO_2_) impacts grain filling and will help to formulate strategies to optimize wheat grain yield and nutritional quality.

## Data Availability Statement

The raw data supporting the conclusions of this article will be made available by the authors, without undue reservation.

## Author Contributions

AG: writing, performing lab, and data analysis. RV and IJ: lab analyses, writing, and review. RS-B: lab analyses and review. RM: supervision, writing, and review. NG: supervision and review. IA: supervision, editing and review. All authors read and approved the present manuscript.

## Conflict of Interest

The authors declare that the research was conducted in the absence of any commercial or financial relationships that could be construed as a potential conflict of interest.
