# Supplementary material for: Differential Flag Leaf and Ear Photosynthetic Performance Under Elevated (CO2) Conditions During Grain Filling Period in Durum Wheat
Source: Front Plant Sci. 2020 Dec 18;11:587958. doi: 10.3389/fpls.2020.587958 (PMC7775369; doi:10.3389/fpls.2020.587958)
Supplement: Supplementary file 1 [file Table_1.DOCX]

| **Supplementary Table S1.** Expression of carbon and nitrogen metabolism genes in the flag leaf and glume in response to growth CO_2_ (A, 400 µmol mol-1, and E, 700 µmol mol-1). The relative content is expressed as log2 of the ratio between the treatment in high CO_2_ and that of ambient CO_2_. Induced or repressed genes vary in blue and red color intensity, respectively, according to color scale (n = 4).  **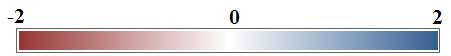** | | | | | | | |
| --- | --- | --- | --- | --- | --- | --- | --- |
|  | | | | | | | |
|  |  |  | **Flag leaf** | |  | **Glummes** | |
| **Acc. No** | **Description** | **Abbreviation** | **E/A** | ***P*** |  | **E/A** | ***P*** |
|  |  |  |  |  |  |  |  |
| **Photosynthetic electron transport chain** | | | | | | | |
| M21352 | Photosystem II protein D1 | D1 | -0.19 | 0.896 |  | -0.50 | 0.080 |
| X75089 | Ferredoxin | Fd | -0.29 | 0.425 |  | -0.31 | 0.273 |
| AJ457980 | Ferredoxin-NADP(H) oxidoreductase | FNR | -0.84 | 0.134 |  | -0.62 | 0.223 |
| M16843 | ATP synthase β-subunit, chloroplastic | pATPasa | 0.07 | 0.563 |  | -0.40 | 0.289 |
|  |  |  |  |  |  |  |  |
| **Calvin-Benson cycle** | | | | | | | |
| AY328025 | Ribulose-1,5-bisphosphate carboxylase oxygenase large subunit | RbcL | -0.56 | 0.524 |  | -1.35 | 0.096 |
| AB020957 | Ribulose-1,5-bisphosphate carboxylase oxygenase small subunit | RbcS | -0.11 | 0.960 |  | -0.73 | 0.066 |
| AJ635203 | Rubisco activase | RBA | -0.63 | 0.396 |  | -1.66 | 0.085 |
| X65540 | Sedoheptulose-1,7-bisphosphatase | SBPasa | -0.14 | 0.573 |  | -0.92 | 0.123 |
|  |  |  |  |  |  |  |  |
| **Carbonic anhydrase** | | | | | | | |
| BE213258 | Carbonic anhydrase, plastidial | CA1 | 0.39 | 0.422 |  | 0.03 | 0.629 |
| TC389217 | Carbonic anhydrase, plastidial | CA2 | 0.45 | 0.523 |  | 1.67 | **0.032** |
| TC393400 | Carbonic anhydrase, plastidial | CA3 | 0.45 | 0.451 |  | -0.73 | 0.143 |
|  |  |  |  |  |  |  |  |
| **Carbohydrate synthesis** | | | | | | | |
| DQ406820 | ADP-glucose-pyrophosphorylase large subunit, plastidial | AGPL | 0.51 | 0.547 |  | -0.07 | 0.876 |
| AF492644 | ADP-glucose-pyrophosphorylase small subunit, plastidial | AGPS | 0.95 | 0.710 |  | -0.76 | 0.198 |
| TC374000 | Fructose-1,6-bisphosphatase, cytosolic | cFBPase | -0.93 | 0.335 |  | -1.55 | 0.130 |
| AF310160 | Sucrose-phosphate synthase 1 | SPS | 0.00 | 0.823 |  | -1.17 | 0.267 |
| EU981912 | Sucrose:sucrose 1-frutosyltransferase | 1SST | -2.96 | **<0.001** |  | -2.51 | 0.060 |
| EU981911 | Sucrose:fructan 6-fructosyltransferase | 6SFT | -0.88 | 0.259 |  | -1.07 | 0.148 |
| AJ564996 | Fructan 1-exohydrolase | 1FEH | -0.47 | 0.182 |  | -2.13 | **0.022** |
|  |  |  |  |  |  |  |  |
| **Glycolysis** | | | | | | | |
| AY974231 | Hexokinase | HXK | -0.45 | 0.283 |  | -1.48 | 0.051 |
| AY130765 | Pyrophosphate-fructose-6-phosphate 1-phosphotransferase | PFP | -3.42 | **0.023** |  | -0.22 | 0.481 |
| AK332778 | Pyruvate kinase | PK | 0.43 | 0.086 |  | -0.54 | **0.043** |
|  |  |  |  |  |  |  |  |
| **Tricarboxylic acid cycle** | | | | | | | |
| GU563379 | Pyruvate dehydrogenase complex E1 component α-subunit | PDC | 0.16 | 0.383 |  | -0.63 | **0.004** |
| AK331640 | NAD-dependent isocitrate dehydrogenase, mithochondrial | IDH | 0.74 | 0.087 |  | -1.00 | **0.037** |
| TC392831 | 2-oxoglutarate dehydrogenase complex E1 subunit | OGDC | -1.16 | 0.080 |  | -1.68 | **0.004** |
|  |  |  |  |  |  |  |  |
| **Nitrogen metabolism** | | | | | | | |
| DQ124209 | Glutamine synthetase, cytosolic | GS1 | 1.61 | 0.033 |  | 0.42 | 0.327 |
| DQ124212 | Glutamine synthetase, plastidial | GS2 | 0.19 | 0.982 |  | -0.42 | 0.476 |
| TC394038 | Putative ferredoxin-dependent glutamate synthase | Fd-GOGAT | -0.34 | 0.422 |  | -1.96 | **0.006** |
| EU885207 | Aspartate aminotransferase, plastidial | cAAT | -0.08 | 0.355 |  | -0.08 | 0.396 |
| EU346759 | Aspartate aminotransferase, cytosolic | pAAT | 0.03 | 0.772 |  | -1.70 | **0.043** |
| AY621539 | Asparagine synthetase | AS | No signal | - |  | -1.35 | 0.059 |
|  |  |  |  |  |  |  |  |
